# Combined mTOR and MEK inhibition is an effective therapy in a novel mouse model for angiosarcoma

**DOI:** 10.18632/oncotarget.25345

**Published:** 2018-05-15

**Authors:** Michelle L. Chadwick, Adam Lane, Dana Thomas, Amanda R. Smith, Angela R. White, Dominique Davidson, Yuxin Feng, Elisa Boscolo, Yi Zheng, Denise M. Adams, Anita Gupta, André Veillette, Lionel M.L. Chow

**Affiliations:** ^1^ Department of Cancer and Cell Biology, University of Cincinnati, Cincinnati, OH, USA; ^2^ Cancer and Blood Diseases Institute, Cincinnati Children’s Hospital Medical Center, Cincinnati, OH, USA; ^3^ Institut de Recherches Cliniques de Montréal, Montréal, Canada; ^4^ Vascular Anomalies Center, Boston Children’s Hospital, Boston, MA, USA; ^5^ Department of Pathology and Laboratory Medicine, Cincinnati Children’s Hospital Medical Center, Cincinnati, OH, USA

**Keywords:** angiosarcoma, mouse model, pre-clinical therapeutics, mTOR, MEK

## Abstract

Angiosarcoma is an aggressive malignancy of vascular origin that occurs *de novo* or in the context of previous cancer therapy. Despite multi-modal aggressive treatment including surgical resection, chemotherapy, and radiation, five-year overall survival remains poor at 35%. Due to its rarity, little is known about its molecular pathology and clinical trials have been extremely difficult to conduct. Development of animal models for rare diseases like angiosarcoma is critical to improve our understanding of tumorigenesis and to test novel treatment regimens. A genetically engineered mouse model for angiosarcoma was generated by conditional deletion of *Trp53*, *Pten*, and *Ptpn12* in endothelial cells. Tumors arising from these mice recapitulate the histology and molecular pathology of the human disease including hyperactivation of the PI3K/mTOR and MAPK signaling pathways. Treatment of tumor-bearing mice with mTOR or MEK inhibitors effectively inactivated signaling and resulted in reduced proliferation and elevated apoptosis leading to tumor regression. The effect of treatment on tumor growth was transient and proliferation was restored after a period of dormancy. However, combined inhibition of mTOR and MEK resulted in profound tumor regression which was sustained for the duration of treatment. These results suggest that angiosarcoma may be effectively treated by this drug combination.

## INTRODUCTION

Angiosarcoma is a rare malignancy of the vascular endothelium accounting for approximately 2% of all soft tissue sarcomas. The approach to treatment is multimodal and includes complete surgical resection where possible followed by chemotherapy and radiation therapy in cases of metastatic disease [1, 2]. Despite aggressive therapy, 5-year overall survival varies from 31% to 43% and is significantly worse with metastatic disease [3–7]. Due to the rarity of the disease and a lack of available model systems, relatively little is known about the molecular pathogenesis of angiosarcoma. In order to develop novel targeted therapeutic regimens for this disease, a greater understanding of the signaling pathways driving tumor growth and models with which to interrogate these pathways are needed.

Although only a few reports have described the driver mutations leading to angiosarcoma development, these reveal the presence of recurrent pathway aberrations. Specifically, mutations leading to activation of the phosphatidylinositol-3′-kinase/mammalian target of rapamycin (PI3K/mTOR) and RAS/mitogen-activated protein kinase (MAPK) pathways have been reported. One study found activating mutations in *PIK3CA* in 40% of tumors [8] while other investigators describe elevated levels of phosphorylated S6 in 100% [9] and phosphorylated 4EBP1 in 88% of angiosarcomas [10], both of which are downstream effectors of mTOR. Importantly, one study demonstrated that treatment with the mTOR inhibitor rapamycin decreases cell proliferation *in vitro* and delayed tumor growth *in vivo* using a xenograft model [9]. Interestingly, a trial of the mTOR inhibitor everolimus in patients with recurrent soft tissue sarcomas reported that the progression free rate was highest in angiosarcoma patients compared to patients with other high-grade sarcomas [11]. Seki *et al.* observed a partial response of an angiosarcoma patient treated with ridaforolimus, another mTOR inhibitor [12]. Mutations in *KRAS* have been found in 13–60% of angiosarcomas as well, leading to activation of both the PI3K/mTOR and MAPK pathways [13–16]. A recent deep sequencing study investigated mutations in the RAS/MAPK pathway comprehensively and found that 53% of angiosarcomas contained hotspot mutations in *KRAS, HRAS, NRAS, BRAF,* or *MAPK1* [17]. To our knowledge, there are no clinical trials of BRAF or MEK inhibitors in patients with angiosarcoma, although one case report described a child with angiosarcoma bearing a *KRAS* mutation who did not respond to the MEK inhibitor trametinib [18]. *TP53* mutations have been noted in 35% to 52% of samples in various studies [16, 17, 19–22]. Moreover, evidence for the involvement of p53 comes from Li-Fraumeni patients who are at increased risk for angiosarcoma [23]. Recently, a genome sequencing effort discovered recurrent mutations in *PTPRB*, a vascular endothelial cell-specific protein tyrosine phosphatase (PTP) that regulates receptor tyrosine kinases (RTKs), in angiosarcoma [16].

PTPN12 is a PTP that has been shown to dephosphorylate RTKs as well as proteins involved in cell migration [24–27]. Developmentally, *Ptpn12* expression is critical as knockout mice die around E10.5 due in part to impaired embryonic vascularization [28]. Several studies have proposed a tumor suppressive role for PTPN12 in various cancers including breast, prostate, colon, kidney, melanoma, and esophageal carcinoma [29–33]. In breast cancer, *PTPN12* loss resulted in a block in apoptosis and increased migration and invasion [34]. Furthermore, in triple negative breast cancer, loss of *PTPN12* has been shown to substitute for amplification of RTKs, and leads to downstream activation of the MAPK pathway. *PTPN12*-null breast cancer cells were also found to have enhanced metastatic potential [24]. Similarly, decreased expression of PTPN12 in colon cancer cell lines resulted in increased cellular migration [31]. Collectively, these data suggest that PTPN12 plays an important role in disease progression in a variety of cancers.

We have developed a novel mouse model for angiosarcoma that encompasses deletion of *Pten*, *Trp53*, and *Ptpn12* in vascular endothelial cells. Deletion of these three genes in concert results in angiosarcoma with complete penetrance and short latency. We demonstrate co-activation of the PI3K/mTOR and MAPK pathways in murine and human tumors and show that *in vivo* concurrent inhibition of mTOR and MEK results in sustained tumor regression.

## RESULTS

### Loss of *Pten*, *Trp53*, and *Ptpn12* results in angiosarcoma

**Figure 1:**
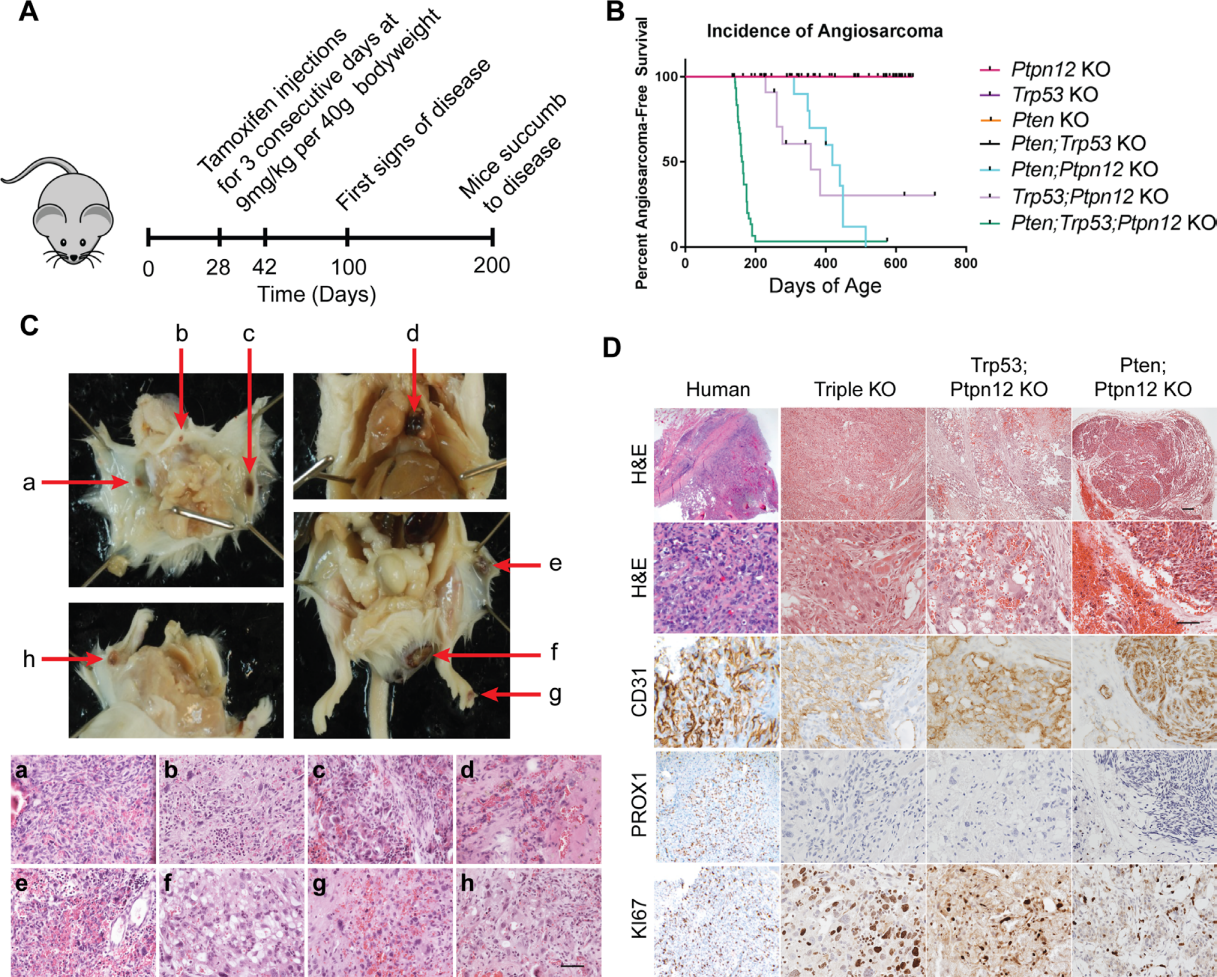
*Ptpn12* deletion leads to angiosarcoma in mice (**A**) Schematic for development of triple knockout angiosarcoma mouse model. (**B**) Kaplan–Meier curve demonstrating incidence of angiosarcoma in the seven mouse lines generated. *Ptpn12* KO *n* = 42, *Trp53* KO *n* = 9, *Pten* KO *n* = 8, *Pten;Ptpn12* KO *n* = 10, *Trp53;Ptpn12* KO *n* = 13, *Trp53;Pten* KO *n* = 10, triple KO *n* = 30. (**C**) Pathology and histology of murine angiosarcomas. Gross dissection of the mouse is shown in the upper panels. Red arrows point to angiosarcomas. Images of the hematoxylin and eosin stained tumors is shown in the lower panels and letter-matched to the gross pictures above (a–h). Scale bar in (h) is 50 µm and applies to all images in the lower panel. (**D**) Histology of human angiosarcoma found in bone compared to the three genotypes of mice that develop angiosarcomas. Scale bar in the top right panel is 100 µm and applies to the first row, scale bar in the panel below is 50 µm and applies to all remaining pictures.

We engineered mice that inducibly delete *Pten*, *Trp53*, and *Ptpn12* using a *GFAP*-CreER mouse driver line. Tamoxifen is administered for three consecutive days between P28 and P44, a time frame chosen to circumvent developmental defects (Figure 1A). We analyzed cohorts of mice with all combinations of single, double and triple gene deletions and found that mice in three of the cohorts [double knockout (DKO) for *Trp53* and *Ptpn12*, DKO for *Pten* and *Ptpn12*, and triple knockout (TKO) for *Trp53*, *Pten* and *Ptpn12*] developed multiple dark cutaneous lesions (Figure 1B), which were easily detectable on the FVB albino background (Figure 1C). Histologic evaluation of the lesions revealed aggressive vascular lesions with anastomosing and solid areas composed of vascular channels lined by pleomorphic endothelial cells, and numerous typical and atypical mitotic figures (Figure 1D). The tumor cells stained strongly for CD31 (a vascular endothelial marker) and were negative for PROX1 (a lymphendothelial marker) consistent with a malignant vascular neoplasm. The resemblance of the histology of the mouse lesion to human angiosarcoma was striking (Figure 1D). Intriguingly, we noted that the three cohorts of mice that developed angiosarcoma all include the deletion of *Ptpn12* (Figure 1B). DKO mice for *Pten* and *Trp53* develop other subcutaneous tumors in addition to high grade glioma, but none develop cutaneous vascular lesions (data not shown). This suggests a critical role for the *Ptpn12* gene in tumor suppression with specificity for angiosarcoma. TKO mice develop tumors with higher penetrance and shorter latency than either of the two DKO genotypes with nearly 100% of mice developing angiosarcomas (Figure 1B). PCR amplification of tumor DNA confirmed Cre-mediated recombination of *Pten*, *Trp53*, and *Ptpn12* (Supplementary Figure 1A).

In order to determine why the *GFAP*-CreER mouse line is driving tumors of endothelial origin, we performed co-immunofluorescence (IF) using antibodies directed against β-galactosidase (which was included as a marker gene on the bicistronic CreER transgene) and CD31. We found that a small percentage of endothelial cells also express β-gal, indicating that CreER is expressed in these cells (Supplementary Figure 1B). Moreover, we crossed the *GFAP*-CreER mouse line with *Rosa*-tdTomato reporter mice and demonstrated Cre-mediated recombination in CD31-positive cells of the skin (Supplementary Figure 1C). Lastly we used an endothelial cell-specific Cre driver, *Tie2-*CreER and found that the TKO combination also developed angiosarcomas, albeit with a broader tissue distribution when compared to the *GFAP-*CreER mice (Supplementary Figure 1D). Tumors in the *GFAP*-CreER mice are generally cutaneous and occur most frequently on the head, extremities, and perianally. Visceral tumors were found in the mediastinum although this occurred in a small percentage of mice (Figure 1C). When tumors from all three genetic combinations were compared, all were found to have a similar immunophenotype with respect to CD31 and PROX1 expression and all demonstrated a high proliferative index as indicated by KI67 staining (Figure 1D). The TKO and *Trp53; Ptpn12* DKO tumors were classified as high-grade angiosarcoma due to the prominent nuclear pleomorphism, atypical mitosis and solid tumor areas observed while tumors from the *Pten; Ptpn12* DKO mice were classified as low-grade with minimal atypia, lack of atypical mitosis and anastomosing morphology.

### Activation of the PI3K/mTOR and MAPK pathways in murine angiosarcoma

**Figure 2:**
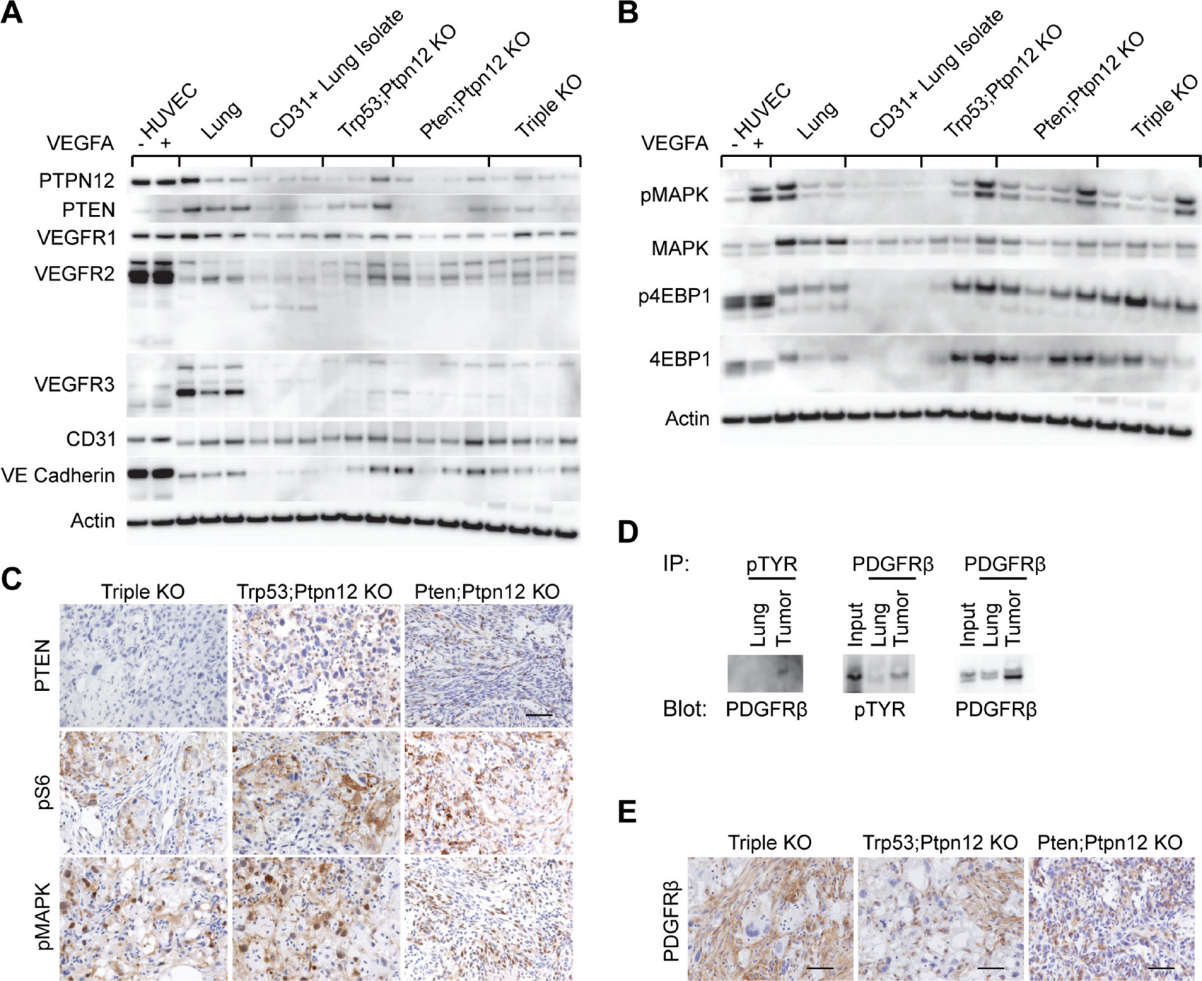
mTOR and MAPK pathways are activated in murine angiosarcoma (**A**) Western blots analyzing different endothelial cell markers in protein lysates prepared from HUVECs untreated (–) or treated (+) with VEGFA, total lung, endothelial cells isolated from lung using CD31 beads, tumors from both *Trp53; Ptpn12* and *Pten; Ptpn12* DKO mice, and tumors from TKO mice. (**B**) Western blots using the same protein lysates as in (A) but investigating the indicated signaling molecules. (**C**) Immunohistochemical staining on tissue sections from a representative tumor from each of the indicated mouse genotypes. Scale bar in the top right panel is 50 µm and applies to all pictures. (**D**) Anti-phosphotyrosine immunoprecipitations (IP) from control lung and TKO angiosarcoma protein lysates were then probed for PDGFR-β (left panel). The reciprocal immunoprecipitation and blotting experiment (middle panel) and total PDGFR-β comparisons (right panel) are shown. The input lane represents 20 µg of protein lysate from the tumor while IPs were performed from 500 µg protein lysate. (**E**) Tumors from the indicated mouse genotypes were stained by IHC for PDGFR-β. Scale bars are 50 µm.

In order to investigate pathways involved in the pathogenesis of murine angiosarcoma we first characterized the expression of known endothelial cell markers in the tumors. CD31 and VEGFR1 are expressed in angiosarcomas at levels comparable to that of CD31+ endothelial cells isolated from mouse lung, while VEGFR2, VEGFR3, and VE-cadherin were relatively overexpressed when compared to the control lung endothelial cells (Figure 2A). These results underscore the vascular nature of these tumors. We then focused on the analysis of the PI3K/mTOR and MAPK pathways as these are known to be regulated respectively by two of the targeted genes, *Pten* and *Ptpn12*. Western blot analysis revealed that phospho-4EBP1 is elevated in all tumors including those that were *Pten* wild-type, suggesting that activation of the mTOR pathway is important for angiosarcoma growth (Figure 2B). Phospho-MAPK is also elevated across all genotypes as expected since loss of *Ptpn12* was common to all tumors. In agreement, we also demonstrated, by immunohistochemistry, high levels of phospho-S6 and phospho-MAPK in tumors of all genotypes (Figure 2C). As expected, PTEN was absent in the *Pten;Ptpn12* DKO and TKO tumors; however, surprisingly, very low levels were also detected in the *Trp53;Ptpn12* DKO tumors (Figure 2A and 2C). STAT3 phosphorylation was evaluated to determine if there was generalized activation of all downstream RTK pathways. However, phospho-STAT3 was not consistently elevated in angiosarcomas relative to endothelial cell controls (data not shown).

We were particularly interested in identifying target(s) of PTPN12 activity critical for angiosarcomagenesis. We first took a candidate approach to test whether RTKs involved in endothelial cell signaling and PTPN12 effectors are phosphorylated in angiosarcomas. Tyrosine phosphorylated proteins were immunoprecipitated from TKO angiosarcoma tumor lysates and blotted for the VEGF receptors (Supplementary Figure 2A–2G). Receptor phosphorylation was either not detected in anti-phosphotyrosine immunoprecipitates (VEGFR1, Supplementary Figure 2A), detected at similar levels compared to control lung tissue (VEGFR2, Supplementary Figure 2B, 2D) or tyrosine phosphorylation was not detected in the reciprocal receptor immunoprecipitation experiment (VEGFR3, Supplementary Figure 2C, 2E). Therefore we decided to use an RTK antibody array to screen for hyperphosphorylated receptor tyrosine kinases in murine angiosarcoma. We found that PDGFR-β was phosphorylated in all three angiosarcoma models and was particularly prominent in *Ptpn12; Pten* DKO tumors in which this was the only RTK with elevated phosphorylation in the array (Supplementary Figure 2H). This finding was verified by anti-phosphotyrosine immunoprecipitation of lysates from TKO tumors followed by western blotting for PDGFR-β as well as by the reciprocal immunoprecipitation experiment (Figure 2D). Furthermore, the presence of high levels of PDGFR-β expression was confirmed by IHC in all three models (Figure 2E). These findings are interesting as PDGFR-β has previously been shown to be a target of PTPN12 [24, 25, 35].

### The mTOR and MAPK pathways can be effectively inhibited using targeted therapies

In order to determine whether these pathways are important for the growth of murine angiosarcomas we treated our most aggressive model, the TKO mice, with small-molecule targeted inhibitors. We used rapamycin, an inhibitor of mTOR and trametinib, an inhibitor of MEK1 and 2, both of which are FDA-approved drugs. To determine the lowest biochemically effective dose of each drug, we treated mice whose tumor measured at least 5mm in its longest diameter daily for five days. Five doses of rapamycin were tested *in vivo*: 25 mg/kg, 10 mg/kg, 5 mg/kg, 3 mg/kg, and 1 mg/kg. Rapamycin effectively inhibited the mTOR pathway down to a dose of 3 mg/kg as assessed by phosphorylation of AKT, S6, and 4EBP1 by Western blot and IHC (Supplementary Figure 3A–3F). Phosphorylation of AKT at S473 is mediated by mTORC2, not mTORC1; however, in some cell lines such as endothelial cells, prolonged exposure to rapamycin can suppress phospho-AKT [36, 37]. At the lowest dose tested, 1 mg/kg, phosphorylation of AKT and 4EBP1 was not significantly diminished; however, S6 phosphorylation remained low (Supplementary Figure 3E). A significant biological effect of treatment with rapamycin was also noted as tumor cell proliferation was decreased and apoptosis, as measured by cleaved caspase-3 was increased (Supplementary Figure 3E, 3F). Moreover all treatment doses tested resulted in regression of tumor size over the five day period of observations (Supplementary Figure 3G). Importantly, treatment with rapamycin appeared to result in an increase in phosphorylation of MAPK suggesting that the tumor cells were attempting to compensate for the loss of proliferative signals from mTORC1 inhibition (Supplementary Figure 3A, 3D, 3E).

The MEK inhibitor, trametinib was tested at three different doses *in vivo*: 3 mg/kg, 2 mg/kg, and 1 mg/kg. We found that phosphorylation of MAPK is effectively inhibited at a dose of 2 mg/kg or greater while partial inhibition was seen at 1 mg/kg by Western blot and IHC (Supplementary Figure 4A–4D). All treatment doses significantly decreased proliferation as measured by KI67 and increased apoptosis as detected by cleaved caspase-3 (Supplementary Figure 4C–4D). As was observed with rapamycin treatment, trametinib treatment at all doses was able to shrink established tumors over the course of five days (Supplementary Figure 4E). Interestingly, the effect on mTOR pathway signaling with MEK inhibition varied at each node. AKT phosphorylation was slightly increased while S6 phosphorylation was diminished and that of 4EBP1 remained relatively unaffected (Supplementary Figure 4A, 4C).

**Figure 3:**
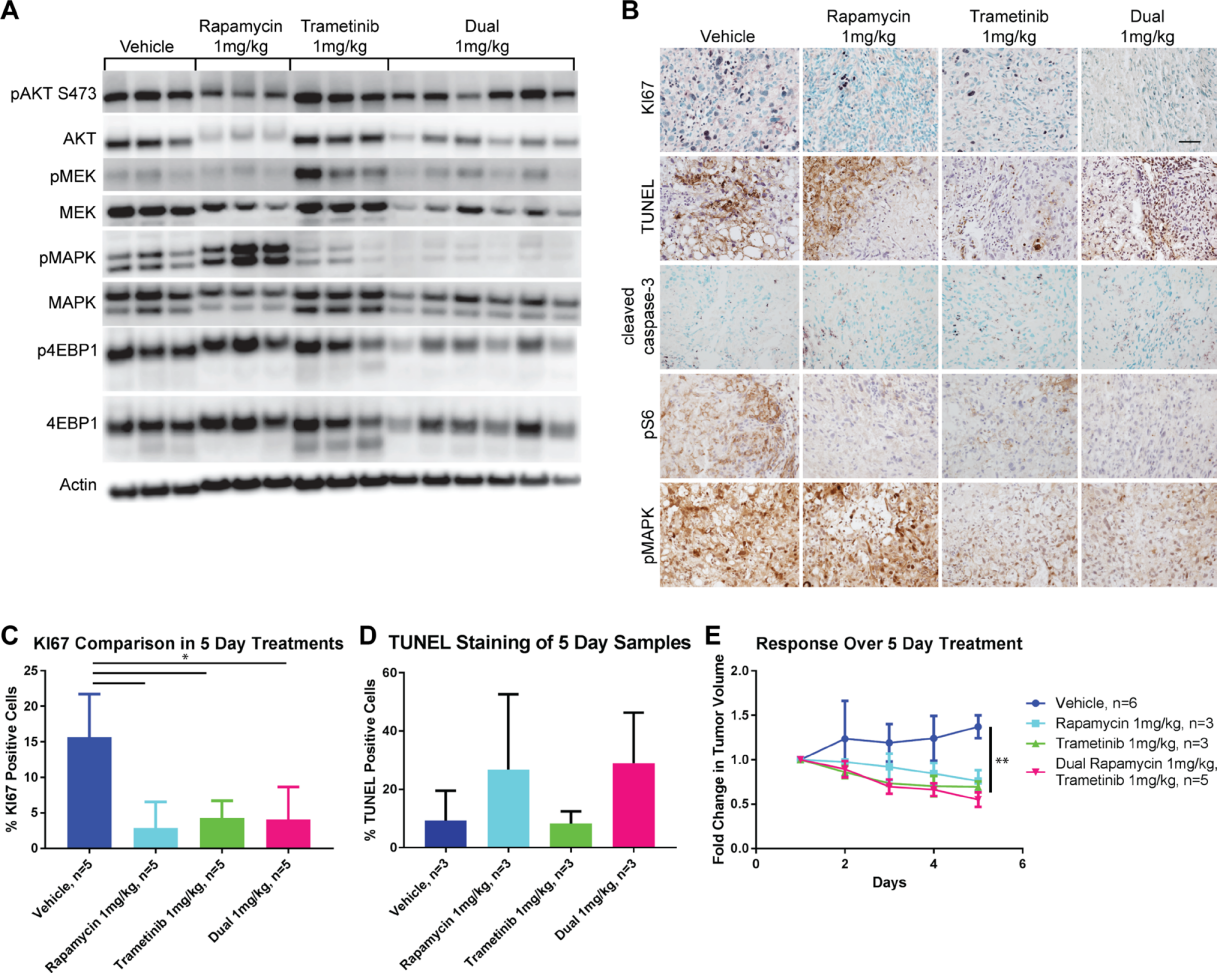
Five-day treatment with trametinib, rapamycin, or a combination of both results in disease regression (**A**) Western blots of signaling molecules on protein lysates prepared from tumors isolated from treated TKO mice comparing vehicle, rapamycin, trametinib or both drugs all administered at a dose of 1 mg/kg. (**B**) IHC demonstrating proliferation (KI67), apoptosis (cleaved caspase-3 and TUNEL), mTOR (pS6), and MAPK (pMAPK) pathway signaling in tumors from the same groups of mice as above. Scale bar in the top right panel is 50 µm and applies to all panels. (**C**) Quantification of KI67 staining from (B). ^*^P ≤ 0.05 applies to all comparisons indicated. (**D**) Quantification of TUNEL staining from (B). (**E**) Comparison of the fold change in tumor volume over the course of the five day treatments. ^**^P ≤ 0.01 applies to all treatment groups compared to vehicle.

We tested combined treatment of both rapamycin and trametinib *in vivo* compared to either drug alone at a dose of 1 mg/kg for 5 days so that we could detect if synergistic or additive effects were present. Dual treatment with these inhibitors resulted in a more profound suppression of signaling in the PI3K/mTOR and MAPK pathways than was seen with either treatment alone at the same dose (Figure 3A). In particular, phospho-4EBP1 was reduced by the dual treatment compared to treatment with rapamycin alone and phospho-MAPK was also diminished compared to treatment with trametinib alone (Supplementary Figure 5A and 5C). Importantly the compensatory hyperphosphorylation of MAPK induced by treatment with rapamycin alone was completely reversed by the addition of trametinib (Supplementary Figure 5C). Similarly increased phosphorylation of both MEK and AKT induced by trametinib was reversed by the addition of rapamycin (Figure 3A and Supplementary Figure 5B). Despite the enhanced biochemical effect on the pathways induced by dual inhibitor treatment, proliferation was similar when compared to either treatment alone (Figure 3B and 3C) and cell death as measured by TUNEL staining was also similar to rapamycin treatment alone (Figure 3B and 3D). Again, there was a significant regression of tumors over the five day treatment period when compared to vehicle treated animals, but this effect was similar to that of either drug treatment alone (Figure 3E).

### Long-term treatment with mTOR and MEK inhibitors is synergistic and results in a sustained anti-tumor response

**Figure 4:**
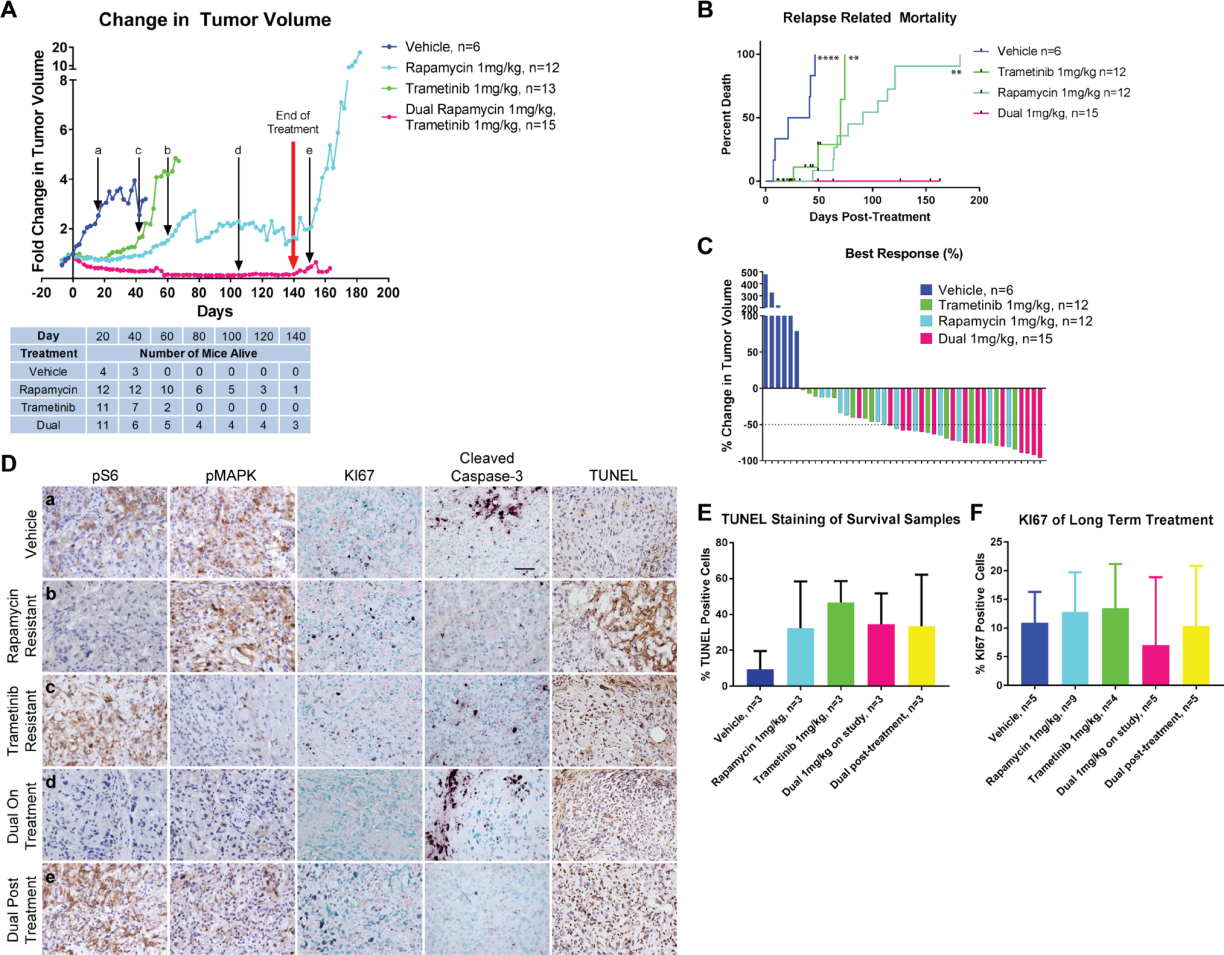
Long-Term Treatment using both rapamycin and trametinib results in sustained disease regression (**A**) Comparison of change in tumor volume in long-term treatments of tumor-bearing TKO mice. The red arrow represents when mice were taken off treatment at 140 days. Black arrows with lower-case letters represent the time points at which samples were analyzed in (**D**). The table below indicates the number of mice remaining from each cohort at the indicated time points. (**B**) Disease-related mortality analyzed by Kaplan–Meier. Non-tumor related deaths are indicated by the black tick marks. Statistical significance beside each curve is compared with the dual drug treatment. ^**^P ≤ 0.01, ^****^P ≤ 0.0001. (**C**) Waterfall plot showing the best responses in drug-treated tumors and maximum growth in vehicle-treated tumors. (**D**) Immunohistochemistry comparing mTOR and MAPK signaling, proliferation and apoptosis at various timepoints. Letters in the lefthand panels correspond to the timepoints when tissue was harvested as delineated in (A). Scale bar in vehicle cleaved caspase-3 panel represents 50 µm and applies to all panels. (**E**) Quantification of TUNEL staining shown in (D). (**F**) Quantification of KI67 staining shown in (D).

In view of the robust biochemical response to rapamycin and trametinib and evidence for tumor regression after only five days of treatment, which was well tolerated by animals, a long-term treatment and survival study was carried out. TKO mice were observed until a single angiosarcoma reached a calculated volume of 100 mm^3^. They were then assigned to one of four treatment arms: vehicle, rapamycin, trametinib, or dual therapy using rapamycin and trametinib at 1 mg/kg each. All treatments were administered daily (seven days a week) continuously until the animal developed morbidity from tumor growth or from the treatment, or until they reached 140 days of treatment whichever came first. As was observed during five-day treatments of mice, all drug treatments were equally effective at inducing tumor regression when compared to the vehicle control (Figure 4A). However, most of the tumors treated with Rapamycin and every tumor treated with Trametinib regrew aggressively between 40 to 80 days of treatment (Figure 4B). In contrast mice treated with both inhibitors experienced tumor regression which was sustained over the 140 day treatment period (Figure 4A). None of the dual drug treated mice succumbed to tumor growth while receiving treatment (Figure 4B), however several animals did die of presumed toxicity of the combined inhibitors (dehydration leading to a moribund state). As a consequence, overall survival of dual inhibitor treated mice was not improved compared to either inhibitor alone (Supplementary Figure 6A). Full necropsy of these animals did not reveal the presence of metastatic disease or any other tumor growth. Importantly, angiosarcomas recurred in mice upon withdrawal of the drugs after 140 days of treatment indicating the persistence of viable tumor cells (Figure 4A).

Response to inhibitor treatment was also assessed by waterfall plot (Figure 4C). This analysis demonstrated that the best responses were achieved in mice who received both rapamycin and trametinib concurrently. Remarkably, 14 of the 15 tumors treated with both inhibitors achieved a partial response (defined as a decrease in tumor volume by at least 50%). This rate of response was significantly greater than that seen in vehicle or trametinib treated mice, and approached statistical significance when compared to rapamycin treatment (Table 1).

**Table 1:** Comparison of best responses to rapamycin, trametinb or combined treatment

Response to Treatment				
	PR (50% Decrease)	<PR	Comparison	*p*-value
Dual	14	1	Dual vs Veh	0.0002
Rapamycin	6	6	Dual vs Rapa	0.054
Trametinib	5	7	Dual vs Tram	0.009
Vehicle	0	6	Rapa vs Veh	0.043
			Rapa vs Tram	0.685
			Tram vs Veh	0.1141

Abbreviations: PR – partial response; Veh – vehicle; Rapa – rapamycin; Tram 013; trametinib.

To determine the cause of tumor recurrence after prolonged treatment with either inhibitor alone, tumors were assessed for pathway activation at various time points indicated in Figure 4A (a–e). Tumors that recurred while on rapamycin treatment maintained complete repression of phospho-S6; similarly, tumors that recurred while on treatment with trametinib exhibited low levels of phospho-MAPK (Figure 4D, Supplementary Figure 6B, 6C). These results suggest that tumor recurrence is not due to loss of the ability of rapamycin or trametinib to suppress their molecular targets, but rather to the development of parallel pathways that support renewed tumor growth. In fact, IHC for KI67 on recurrent tumors revealed brisk levels of proliferation similar to levels seen in vehicle treated tumors while proliferation remained low in dual inhibitor treated tumors. Elevated apoptosis persisted in treated tumors as detected by TUNEL staining (Figure 4D–4F). In tumors treated for five days with rapamycin or trametinib, hyperactivation of the uninhibited pathway was noted (Figure 3A, 3B and Supplementary Figure 5B, 5C). In the long-term treatments, some tumors continued to exhibit similar hyperphosphorylation however this was not consistently seen in all tumors. Therefore, we assessed other RTK downstream effector pathways that could be targets for PTPN12-mediated regulation driving tumor growth. Phosphorylation of STAT3 has been implicated in the growth of angiosarcoma cells [38] however neither rapamycin nor trametinib treated tumors were found to have consistently elevated levels of phospho-STAT3 Y705 (Supplementary Figure 6B, 6C). We also assessed the phosphorylation of focal adhesion kinase (FAK), a known target of PTPN12 [39] and found that recurring tumors in trametinib treated mice did not have an increase in FAK phosphorylation (Supplementary Figure 6C). While other growth promoting pathways may be involved in tumor regrowth in mice treated with a single agent, it is possible that tumor proliferation is driven by a subtle shift in signaling towards the mTOR pathway in tumors treated with trametinib and towards the MAPK pathway in tumors treated with rapamycin, emphasizing the importance of simultaneous inhibition of both pathways in order to achieve a sustained response.

### Activation of the PI3K/mTOR and MAPK pathways in human tumors of vascular origin

**Figure 5:**
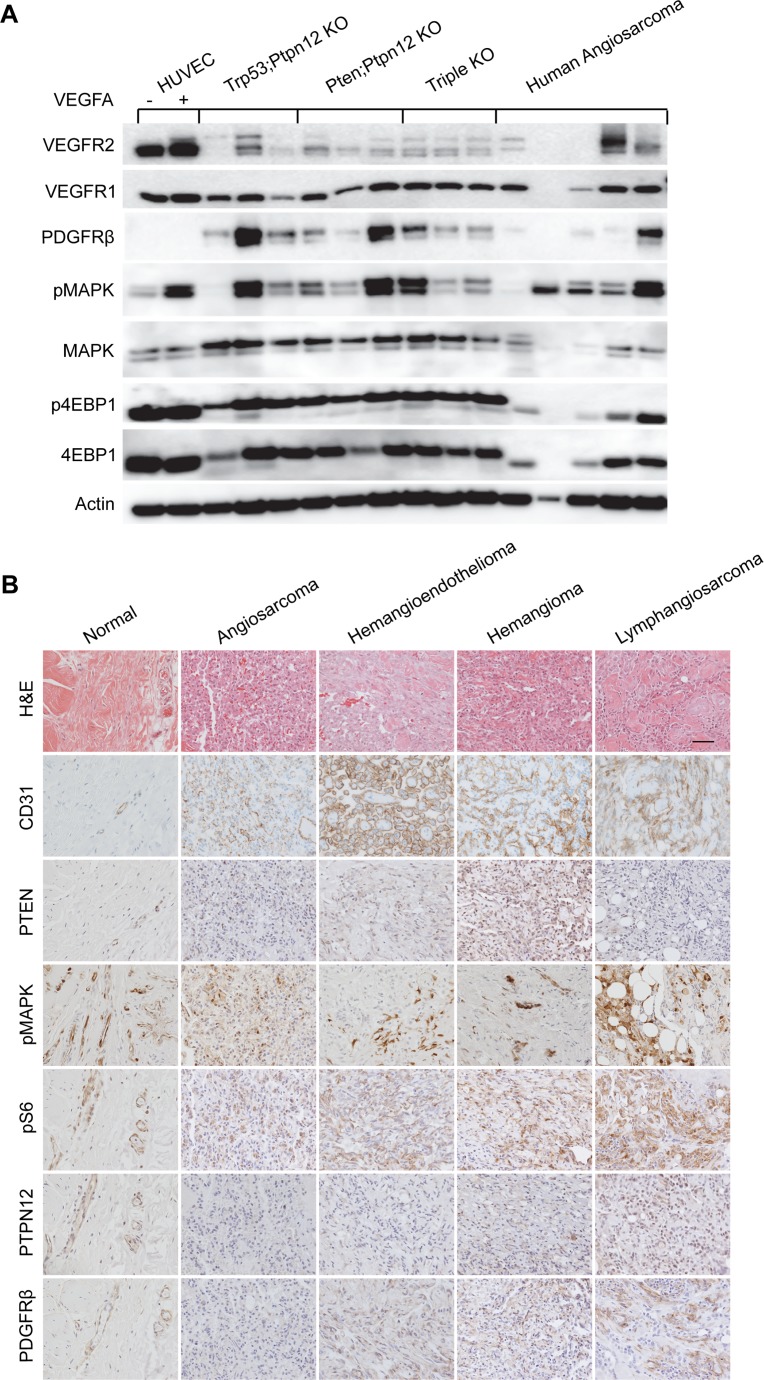
Murine angiosarcomas closely mimic human disease (**A**) Western blot analysis of protein lysates prepared from HUVECs treated (+) or untreated (–) with VEGFA, angiosarcomas from all three mouse genotypes, and a panel of human angiosarcomas. (**B**) IHC analysis of normal human skin blood vasculature compared with the indicated endothelial cell tumor types. Scale bar in top right panel is 50 µm and applies to all panels.

Five snap frozen aliquots of angiosarcoma were obtained from the pathology biobank at Cincinnati Children’s Hospital Medical Center. Signaling in these tumors was compared to tumors from all three mouse genotypes as well as to HUVECs with and without stimulation by VEGFA (Figure 5A). VEGFR2 was expressed in the human angiosarcoma samples in three of the tumors while VEGFR1 and PDGFR-β were detected in four each. Both the mTOR and MAPK pathways were activated as demonstrated by phospho-4EBP1 and phospho-MAPK respectively (Figure 5A). Anti-phospho-tyrosine western blotting demonstrated an overlapping pattern of tyrosine phosphorylated proteins in the human angiosarcoma samples compared to tumors from our mouse models (Supplementary Figure 7).

A panel of human tumors of vascular origin were also analyzed by IHC (Figure 5B). The endothelial origin of all of the tumors was confirmed by showing immunoreactivity for CD31. Interestingly, PTEN expression was largely absent in high-grade tumors (angiosarcoma and lymphangiosarcoma) while phosphorylation of S6 and MAPK was present in all tumor types. Further, expression of the PTPN12 protein was also very low in this panel of tumors when compared to normal blood vessels. PDGFR-β was not detectable by IHC in the angiosarcoma sample but was found in other tumors of vascular origin. Collectively, these results suggest that at least a subset of human angiosarcomas could benefit from treatment that we find to be effective against our murine angiosarcoma model. Furthermore, other tumors of endothelial cell origin have similar pathway activation and may also respond to this treatment regimen.

## DISCUSSION

We developed a novel and robust genetically engineered mouse model for angiosarcoma through the combined deletion of *Pten, Trp53,* and *Ptpn12.* Conditional deletion of all three genes results in aggressive cutaneous tumors in mice that develop rapidly and with virtually 100% penetrance. The vascular origin of these tumors was confirmed by immunohistochemical staining of tumors for vascular markers, confirmation of rare endothelial cells in the skin targeted by the *GFAP*-CreER driver line and recapitulation of the angiosarcoma phenotype using a more specific vascular endothelial driver, *Tie2*-CreER. Notably, the vascular tumors from our models did not express PROX1 and did not have associated lymphatic effusions, indicating that these tumors were most likely angiosarcomas rather than lymphangiosarcomas. Recently, investigators used a different endothelial mouse driver line to conditionally ablate *Tsc1* and to activate mTORC1 signaling in the vascular endothelium [40]. These mice primarily develop cutaneous and hepatic lymphangiosarcoma. Moreover establishment of an autocrine VEGF signaling loop was shown to contribute to tumor growth in this model. The differences in tumor phenotype between the current report and the study of Sun *et al.* are likely multifactorial. First, different populations of endothelial cells and/or progenitors are targeted by the different mouse driver lines used. Second, while mTORC1 signaling in endothelial cells was induced in both studies, this was accomplished by inactivating different nodes of the pathway which is likely to result in a different pattern of secondary signals. It is interesting to note that in our model, conditional deletion of *Pten* alone did not result in any tumors [41]. Finally, unlike the previous report, we have combined activation of mTORC1 signaling with other oncogenic drivers specific to angiosarcomagenesis. This combination, and in particular inactivation of *Ptpn12*, was highly associated with the angiosarcoma phenotype in mice. The comparative study of these two valuable animal models may elucidate critical differences in the biology and cell of origin for these two related vascular tumors. They may also help to define the appropriate molecular targets for therapeutic intervention of these two entities, which are currently treated with approaches resulting in overall poor outcomes.

As previously mentioned, one striking finding of this study was the association of *Ptpn12* recombination and inactivation in endothelial cells with the development of angiosarcoma. Vascular tumors were not observed in other targeted combinations of tumor suppressor deletion using the same Cre driver line [41] including substitution of *Ptpn12* deletion in this model by activation of MAPK signaling through the expression of a *BRAF* mutant allele (BRAF V600E; ARS and LMLC, unpublished data). These results suggest that the PTPN12 protein is a tumor suppressor for endothelial cells and that it regulates pathways that govern malignant progression to angiosarcoma. We analyzed the activation status of known PTPN12 substrates and RTKs expressed in vascular endothelial cells and several were found to be tyrosine phosphorylated in angiosarcoma. However PDGFR-β was the only substrate whose phosphorylation was reliably elevated when compared to control tissue and it was the only RTK that was phosphorylated in angiosarcomas from all three genetic crosses. While PDGFR-β is predominantly a marker of pericytes [42], there are several reports suggesting that its expression can be induced in endothelial progenitor cells particularly in the context of proliferation in response to vascular injury [43, 44]. Additional studies will focus on elucidating other critical substrates of PTPN12 loss in angiosarcoma which could lead to the identification of novel targets for therapy.

PTPN12 is a candidate tumor suppressor gene in several human cancers including breast cancer, hepatocellular carcinoma, oral squamous cell carcinoma, colon cancer, esophageal cancer, non small cell lung cancer, nasopharyngeal carcinoma, and glioblastoma [24, 31, 33, 35, 45–49], however it has not been studied in the context of human angiosarcoma. Recently another PTP, *PTPRB*, was found to be mutated in 26% of angiosarcomas [16]. PTPRB, which is also referred to as VE-PTP, has been shown to dephosphorylate and to regulate the activity of TIE2 and VEGFR2 in endothelial cells [50, 51]. TIE2 appears to be an especially sensitive substrate in mature endothelium as targeted inhibition of PTPRB was demonstrated to activate receptor signaling and to reduce neovascularization associated with macular degeneration and diabetic retinopathy [52]. However, the substrates of PTPRB in endothelial progenitor cells have not been elucidated nor have they been studied in the context of angiosarcoma. Our results suggest that PTPN12 may phenocopy the tumor suppressor role of PTPRB in murine endothelial cells. Alternatively, PTPRB may be one of several PTPs involved in the pathogenesis of endothelial cell-derived tumors. It should be noted that potentially inactivating mutations of other PTP-encoding genes were found with low frequency in whole exome/genome sequencing of tumor DNA [16, 17]. While *PTPN12* was not one of these genes, the number of angiosarcoma genomes that have been sequenced to date remains small.

The contribution of RTK signaling to the pathogenesis of angiosarcoma was suggested with the finding of amplifications and mutations in the genes encoding the VEGFR2 and VEGFR3 [53, 54]. The description of PTPRB mutations in angiosarcoma not only reconfirms the importance of RTK signaling in this tumor, but also raises the possibility that a broader range of RTKs expressed (or misexpressed) in endothelial cells may be derepressed and contribute to oncogenesis. We confirmed that PDGFR-β is phosphorylated in these murine angiosarcomas, however activation of other RTKs was also suggested by our screening RTK array experiment (Supplementary Figure 2H). These findings have important implications in the treatment of patients with angiosarcoma. Contemporary clinical trials have focused on targeting RTKs involved in angiogenesis with monoclonal antibodies such as bevacizumab [55] or targeted kinase inhibitors such as sorafenib and pazopanib [56–58]. Overall, the clinical benefit of these treatments has been short-lived with progression free survival (PFS) ranging from 1.8 to 3.8 months in these studies. This is comparable to the PFS of 4 months reported in a Phase II trial of paclitaxel alone [59]. Furthermore, in a randomized Phase II trial of paclitaxel compared to paclitaxel and bevacizumab, no improvement in PFS was seen with the addition of the antiangiogenic agent to chemotherapy [60]. The lack of overall response in these clinical trials may therefore be due to redundancy of RTKs expressed and activated in angiosarcoma and/or direct mutational activation of downstream effectors that are not dependent on RTK activity. Our work suggests that a more robust and durable response to therapy may be obtained by targeting two primary downstream effector pathways of RTKs.

We investigated the activation status of the PI3K/mTOR and MAPK pathways in murine angiosarcoma and found both to be active in all three genetic combinations. Moreover, in concordance with other studies, these pathways were also found to be activated in human angiosarcoma as well as other vascular tumors. Therefore we investigated the effect of inhibition of these pathways separately and in combination *in vivo* in our mouse model using the FDA-approved drugs rapamycin, an mTOR inhibitor and trametinib, a MEK inhibitor. First we determined that administration of these drugs at doses relevant to clinical applications effectively inhibited the intended targets. We were surprised that five-day courses of either drug resulted in significant regression of tumor volume. However tumor resistance to monotherapy was heralded by the observation of reciprocal pathway hyperactivation. Indeed, long term monotherapy resulted in inevitable tumor regrowth. We found that combined therapy was not only able to prevent reciprocal pathway hyperactivation but resulted in a more profound and sustained tumor regression than either drug alone.

A significant limitation of our study is the mortality of mice due to toxicity from the combined agents. To mitigate these effects, we defined the minimally effective biochemical dose of each agent independently, then decreased each drug by one dose level in combination (1 mg/kg each). We conducted a further *in vivo* dose de-escalation study to 0.5 mg/kg of each drug in combination which is 25% and 17% respectively of the minimally effective biohemical dose of trametinib and rapamycin in this model (data not shown). Remarkably, this combination also resulted in sustained anti-tumor activity comparable to higher doses, but still caused treatment limiting toxicity in mice. Nevertheless, it should be noted that combinations of MEK and mTOR inhibitors have been studied in early phase clinical trials [61, 62]. Both trials were designed to achieve doses as close to the recommended single agent dose as possible. One trial was a Phase I dose-finding study of the combination of trametinib and the rapalog, everolimus and was deemed to have failed due to the inability of patients to tolerate pre-defined optimal doses [62]. However, no pharmacodynamic analyses of tumor tissue were conducted to look for target inhibition. Importantly, no dose-limiting toxicities were reported at the starting dose level which corresponded to 25% of the single agent dose for trametinib and 50% for everolimus. In the second trial, which was a Phase II trial of the MEK inhibitor, selumetinib given at 67% if the single agent dose and the rapalog, temsirolimus given at 100% (subsequently reduced to 80%), less than 10% of patients who received the combined treatment withdrew from the study due to toxicity [61]. Again no pharmacodynamic studies in tumor tissue were conducted in this trial. The dramatic response to combined *in vivo* mTOR and MEK inhibition which we have observed in our genetically engineered animal model for angiosarcoma should renew efforts to design clinical trials for patients with this disease. We suggest that these trials should incorporate tumor pharmacodynamic analyses in order to determine target inhibition, a dose de-escalation scheme, and adequate supportive care to mitigate the incidence of mucosal inflammation and stomatitis which were the most frequent grade 3 toxicities noted in previous clinical trials of combined therapy.

## MATERIALS AND METHODS

### Mice

Mice used in this study were backcrossed to the FVB/NJ background for at least six generations unless otherwise noted below. The *GFAP*-CreER, *Trp53* floxed, *Pten* floxed and *Ptpn12* floxed mice have been previously reported [63–66]. The *Rosa*-tdTomato reporter mouse (Jackson Laboratory, Bar Harbor, ME, USA; stock no. 007909) was backcrossed to FVB/NJ for three generations for these experiments. The endothelial specific *Tie2*-CreER^T2^ mouse will be described elsewhere (Y.F. and Y.Z., submitted) and was maintained on the C57Bl6 background. Tamoxifen (Sigma-Aldrich, St. Louis, MO, USA) was prepared and administered to mice as previously described [41]. Mice were observed daily and euthanized following NIH guidelines. All mouse experiment were conducted after review and approval from the Animal Care and Use Committee of Cincinnati Children’s Hospital Medical Center. Tissue collection and preparation has been previously described [41].

### Drug treatments

For rapamycin (LC Laboratories, Woburn, MA, USA) 25 mg/kg dosing, a stock solution of 50 mg/ml in dimethyl sulfoxide (DMSO; Sigma-Aldrich) was made. Prior to treatment this solution was diluted to a working solution of 2.5 mg/ml in 5.2% Tween 80 (Sigma-Aldrich) in ddH2O. For all other doses, a stock solution of 10 mg/ml was used and diluted to working solutions of 1, 0.5, 0.3, and 0.1 mg/ml. For trametinib (LC Laboratories) dosing, a stock solution of 10 mg/ml in DMSO was made and diluted to working solutions of 0.3, 0.2, and 0.1 mg/ml in 5.2% Tween 80 prior to use. All stock solutions were aliquoted and stored at –20° C for up to one week. Working solutions were prepared daily and mice were treated with 100 µl/10 g body weight to achieve the desired dose. Rapamycin was administered through intraperitoneal injection while trametinib was given via oral gavage. Vehicle solutions (DMSO with no drug) were prepared and administered in the same manner as the drug treatments. Mice treated with rapamycin were euthanized 2 hours after the last dose, trametinib treated mice 4 hours after the last dose, and dual treated mice 3 hours after the last dose. Tumors were measured by calipers three times weekly and mice were weighed once weekly throughout treatment.

### Endothelial cell isolation

Sheep anti-Rat magnetic Dynabeads (Thermo Fisher Scientific, Waltham, MA, USA), 200 µl/sample, were couple to 5 µl of anti-CD31 antibody (BD Biosciences, Franklin Lakes, NJ, USA; 553370) according to the manufacturer’s instructions. Cells were obtained from the lungs of adult (>8 week old) FVB mice. The lungs were dissected, minced, and incubated in DMEM media with Collagenase Type 1 (Thermo Fisher Scientific) at 2 mg/ml for 1 hour at 37° C. The tissue was passed through a 70 micron filter (Greiner Bio-One, Monroe, NC, USA) and centrifuged for 5 min. at 200*g*. The cell pellet was resuspended in 2 ml phosphate buffered saline (PBS) + 0.1% bovine serum albumin (BSA) and added to the anti-CD31 antibody-conjugated Dynabeads. The cells were incubated on a rotator for 15 min. at room temperature then washed three times in PBS + 0.1% BSA and the resulting Dynabead attached cells used for protein lysate preparation.

### Western blot

Protein lysates from frozen tissues were prepared and Western Blots performed as previously described [41] using 10 to 25 µg total protein depending on the blot. Antibodies used include the following from Cell Signaling Technologies (Danvers, MA, USA; all of them used at 1:1000): anti-pAKT S473 (9271), anti-AKT (4685), anti-p4EBP1 (9459), anti-4EBP1 (9452), anti-pMAPK (9101), anti-MAPK (9102), anti-PTEN (9559), anti-FAK (3285), anti-VEGFR2 (2479) and anti-PDGFR-β (3169). Other antibodies were also used at 1:1000 except where indicated: anti-PTPN12 (Abcam, Cambridge, MA, USA; ab76492), anti-VEGFR1 (Abcam; ab32152), anti-VEGFR3 (Thermo Fisher Scientific; PA5-16871), anti-CD31 (Abcam; ab28364), anti-actin (Sigma-Aldrich; A5441, 1:10000), anti-VE-cadherin (Santa Cruz, Dallas, TX, USA; sc-6458), anti-pTyr 4G10 (Millipore, Billerica, MA, USA; 05-321), anti-pFAK (Sigma; F7926). Blots were imaged on a BioSpectrum Imaging System (UVP, Upland, CA, USA) and quantification of bands was performed using the instrument software.

### Immunohistochemistry/immunofluorescence

Immunohistochemistry (IHC) and immunofluorescence (IF) were performed as previously described [41, 64]. IHC staining was performed on 5 µm thin paraffin sections while IF staining was done on 12 µm frozen sections. Antigen retrieval was performed using citrate buffer and microwave heating. The antibodies used in IHC were anti-CD31 (1:100), anti-PROX1 (gift from M. Nakafuku, CCHMC; 1:2000), anti-KI67 (Leica Microsystems, Bannockburn, IL, USA; NCL-Ki67p, 1:4000), anti-PTEN (1:1000); anti-pS6 (1:5000), anti-pMAPK (1:400), anti-PDGFR-β (1:200), anti-cleaved caspase-3 (BD Biosciences; 559565, 1:500) and anti-PTPN12 (1:1000). The appropriate biotinylated secondary antibodies (1:200) were used in conjunction with horseradish peroxidase-conjugated streptavidin (Elite ABC; Vector Labs, Burlingame,CA, USA), revealed with DAB or VIP substrate (Vector Labs) and counterstained with hematoxylin or methyl green respectively (Vector Labs). The antibodies used in IF were anti-β-galactosidase (Aves Labs, Tigard, OR, USA; BGL-1040, 1:1000) and anti-CD31 (1:100). β-galactosidase staining was revealed with a donkey anti-chicken antibody conjugated to Alexa 488 (Jackson ImmunoResearch, West Grove, PA, USA; 1:200). CD-31 was detected using the Tyramide Amplification method [67]. Briefly, after incubation with the primary antibody, slides were incubated with biotinylated goat anti-rat secondary antibody, followed by Elite ABC (Vector Labs). This was followed by reaction with biotinylated tyramide and revealed with streptavidin conjugated to Cy3 or Alexa 488 (Jackson ImmunoResearch; 1:200). Terminal deoxynucleotidyl transferase dUTP nick-end labeling (TUNEL) assay was performed with the ApopTag Kit (Millipore) according to the manufacturer’s instructions.

### Immunoprecipitation

Immunoprecipitation (IP) experiments were performed using Dynabeads Protein A (Thermo Fisher Scientific) conjugated to the indicated antibodies according to the manufacturer’s instructions. For IP of tyrosine-phosphorylated proteins the P-Tyr-1000 antibody was used (Cell Signaling Technologies; 8954). IP was performed using 500 µg of protein from CD31+ lung endothelial cells, whole lung, and tumor lysates. Recovered proteins were then run on Western Blots as described above and probed for the indicated proteins.

### RTK array

RTK array experiments were performed according to the manufacturer’s instructions (R&D Systems, Minneapolis, MN, USA; ARY014) using 500 µg of protein lysate on each array.

### Polymerase chain reactions

Recombination of floxed alleles was detected by polymerase chain reaction (PCR) using the following oligonucleotides: for the *Ptpn12* gene 5′ GCTCCAAGGTTAAATGCCC 3′ and 5′ TCTATGCTGTGTAACTAGC 3′; for the *Pten* gene 5′ TAGTTGGAGTCACCAGGATG 3′ and 5′ AAGAGTCAAACAATGGCAAGC 3′; and for the *Trp53* gene 5′ CACAAAAACAGGTTAAACCCAG 3′ and 5′ GAAGACAGAAAAGGGGAGGG 3′. Expected band sizes for the recombined products were 290 bp, 900 bp and 600 bp respectively.

### Statistics

Statistical analysis was performed using GraphPad Prism software. Quantitation of Western blots and 5-day treatment tumor volumes utilized two-tailed Student’s *t* test. Fisher’s Exact Test was used to compare response rate of different treatment groups following long-term treatment. Survival analyses were performed using the method of Kaplan and Meier. Unless otherwise indicated, statistical significance was as follows: ^*^P ≤ 0.05, ^**^P ≤ 0.01, ^***^P ≤ 0.001, ^****^P ≤ 0.0001.

### Study approval

Use of human material for this study was approved by the Institutional Review Board at CCHMC.

## SUPPLEMENTARY MATERIALS FIGURES AND TABLE



## Abbreviations

BSA: bovine serum albumin
DKO: double knockout
DMEM: Dulbecco modified eagle medium
DMSO: dimethyl sulfoxide
FDA: Food and Drug Administration
HUVEC: human umbilical vein endothelial cells
IF: immunofluorescence
IHC: immunohistochemistry
IP: immunoprecipitation
KO: knockout
MAPK: mitogen-activated protein kinase
mTOR: mammalian target of rapamycin
mTORC1: mammalian target of rapamycin complex 1
NIH: National Institutes of Health
PBS: phosphate buffered saline
PCR: polymerase chain reaction
PFS: progression free survival
PI3K: phosphatidylinositol-3′-kinase
PTP: protein tyrosine phosphatase
RTK: receptor tyrosine kinase
TKO: triple knockout
TUNEL: terminal deoxynucleotidyl transferase dUTP nick-end labeling
