# Supplementary material for: Combined mTOR and MEK inhibition is an effective therapy in a novel mouse model for angiosarcoma
Source: Oncotarget. 2018 May 15;9(37):24750–65. doi: 10.18632/oncotarget.25345 (PMC5973867; doi:10.18632/oncotarget.25345)
Supplement: Supplementary file 1 [file oncotarget-09-24750-s001.pdf]

Combined mTOR and MEK inhibition is an effective therapy in a novel mouse model for angiosarcoma

SUPPLEMENTARY MATERIALS

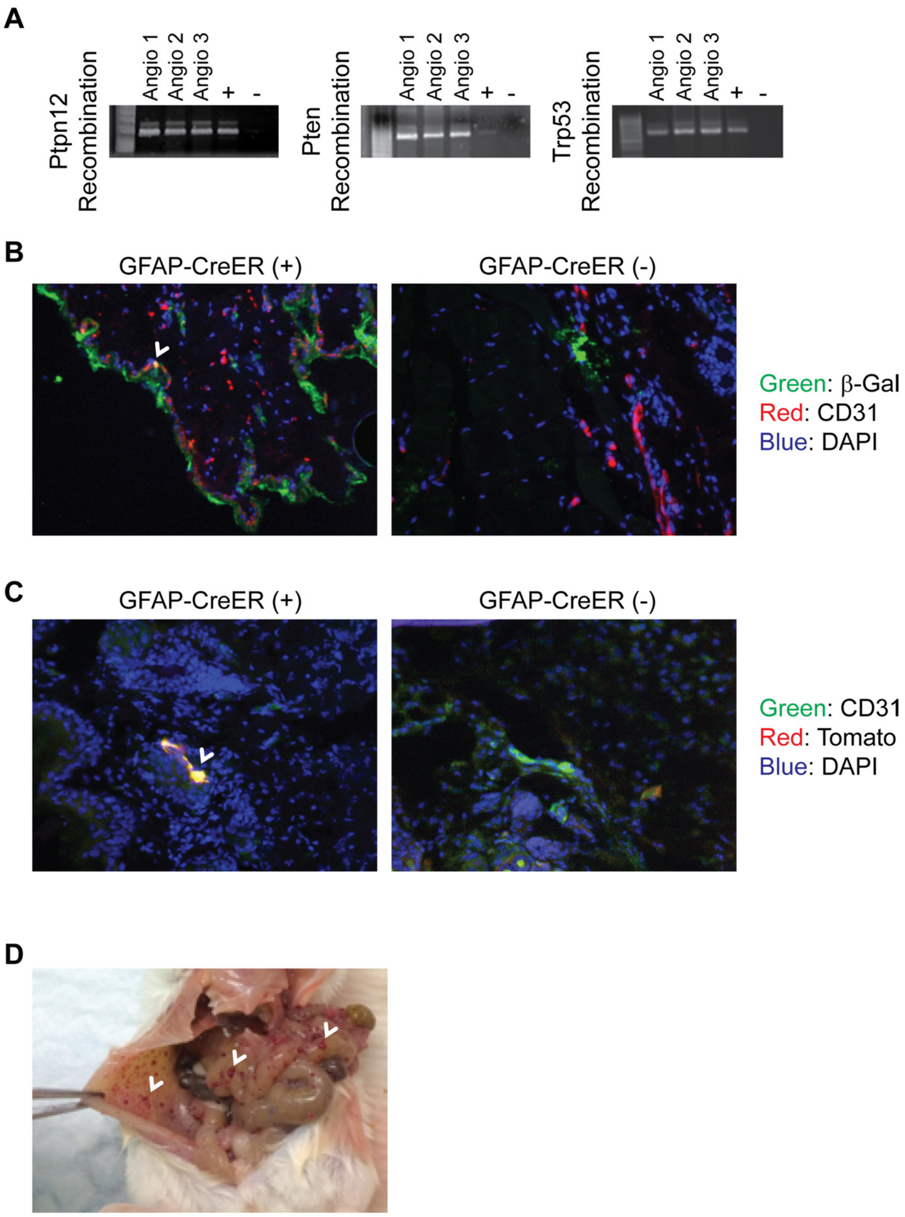

Supplementary Figure 1: Mouse model deletes genes in a subset of endothelial cells leading to angiosarcoma. (**A**) PCR for the recombination products of *Ptpn12, Pten,* and *Trp53* show the predicted amplification of bands corresponding to recombination of all three genes in the TKO tumors. (**B**) Double immunofluorescence (IF) demonstrating colocalization of β-gal (green) and CD31 (red) in occasional cells (white arrowhead) in the skin of *GFAP*-CreER (+) mice but not in *GFAP*-CreER (–) mice indicating that CreER is expressed in a subset of endothelial cells in the skin. (**C**) Double IF demonstrating Cre-mediated recombination in skin endothelial cells of *GFAP*-CreER (+) mice crossed to the *Rosa*-tdTomato reporter mouse. TdTomato fluorescence (red) overlaps with CD31 expressing cells (green) in *GFAP*-CreER (+) mice (white arrowhead) confirming Cre activity in skin endothelial cells. (**D**) Gross dissection of the thorax and abdomen of *Tie2*-CreER^{T2}-driven TKO mice. The white arrowheads indicate angiosarcomas studding the peritoneum and intestinal surfaces.

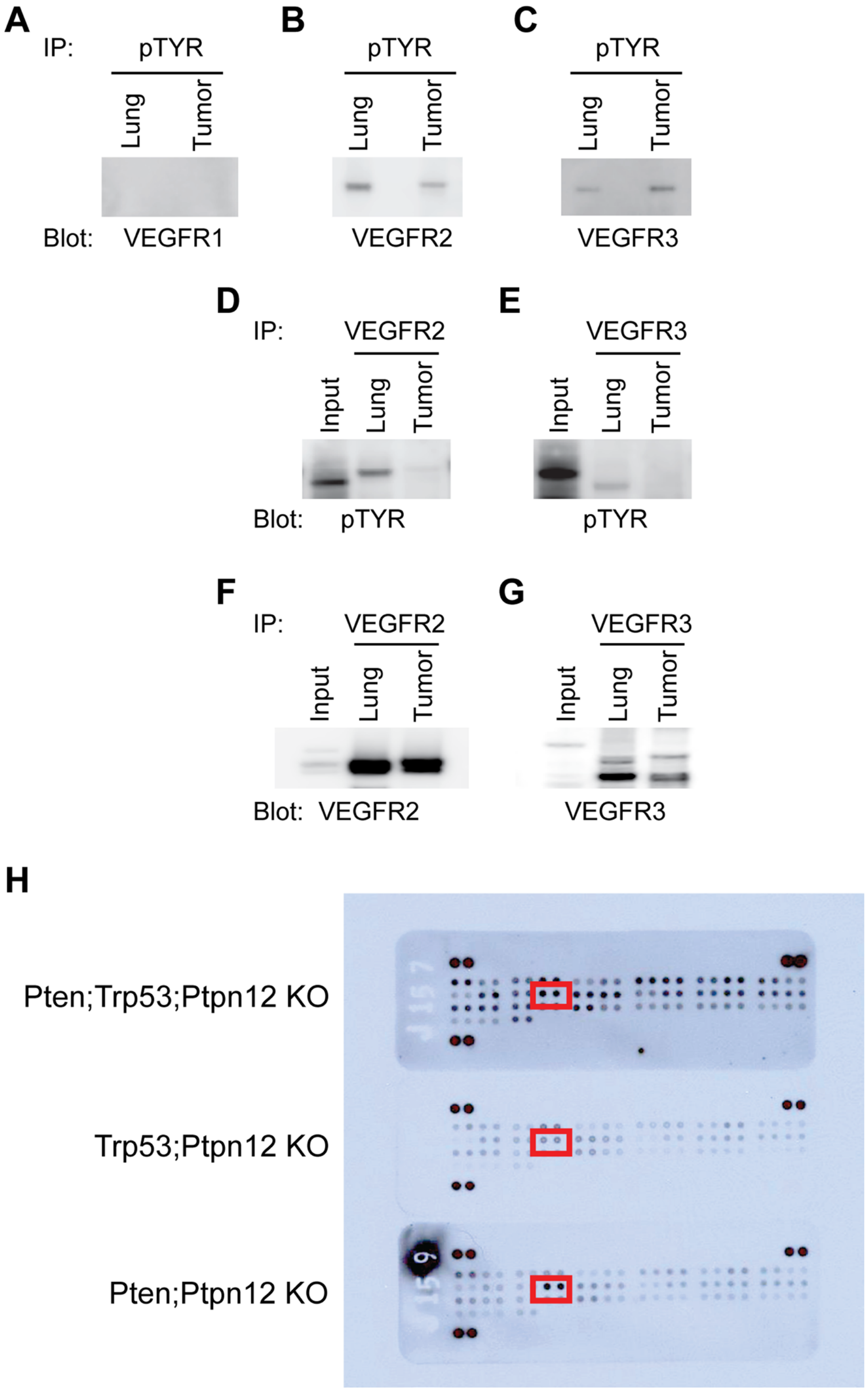

Supplementary Figure 2: PDGFR-β is phosphorylated in angiosarcoma while the VEGF receptors are not. Immunoprecipitations were performed with the P-Tyr-1000 antibody followed by blotting with anti-VEGFR1 (**A**), anti-VEGFR2 (**B**), and anti-VEGFR3 (**C**) antibodies. Immunoprecipitations were then performed with anti-VEGFR2 (**D**) and anti-VEGFR3 (**E**) antibodies followed by blotting with the 4G10 anti-phosphotyrosine antibody. These blots were also probed with anti-VEGFR2 (**F**) and anti-VEGFR3 (**G**) antibodies to verify successful IP. (**H**) RTK arrays were incubated with protein lysates from angiosarcomas isolated from the indicated mice. Spots corresponding to the PDGFR-β protein are indicated with red boxes. The complete index of all RTKs represented on the array can be found in the Supplementary Table 1.

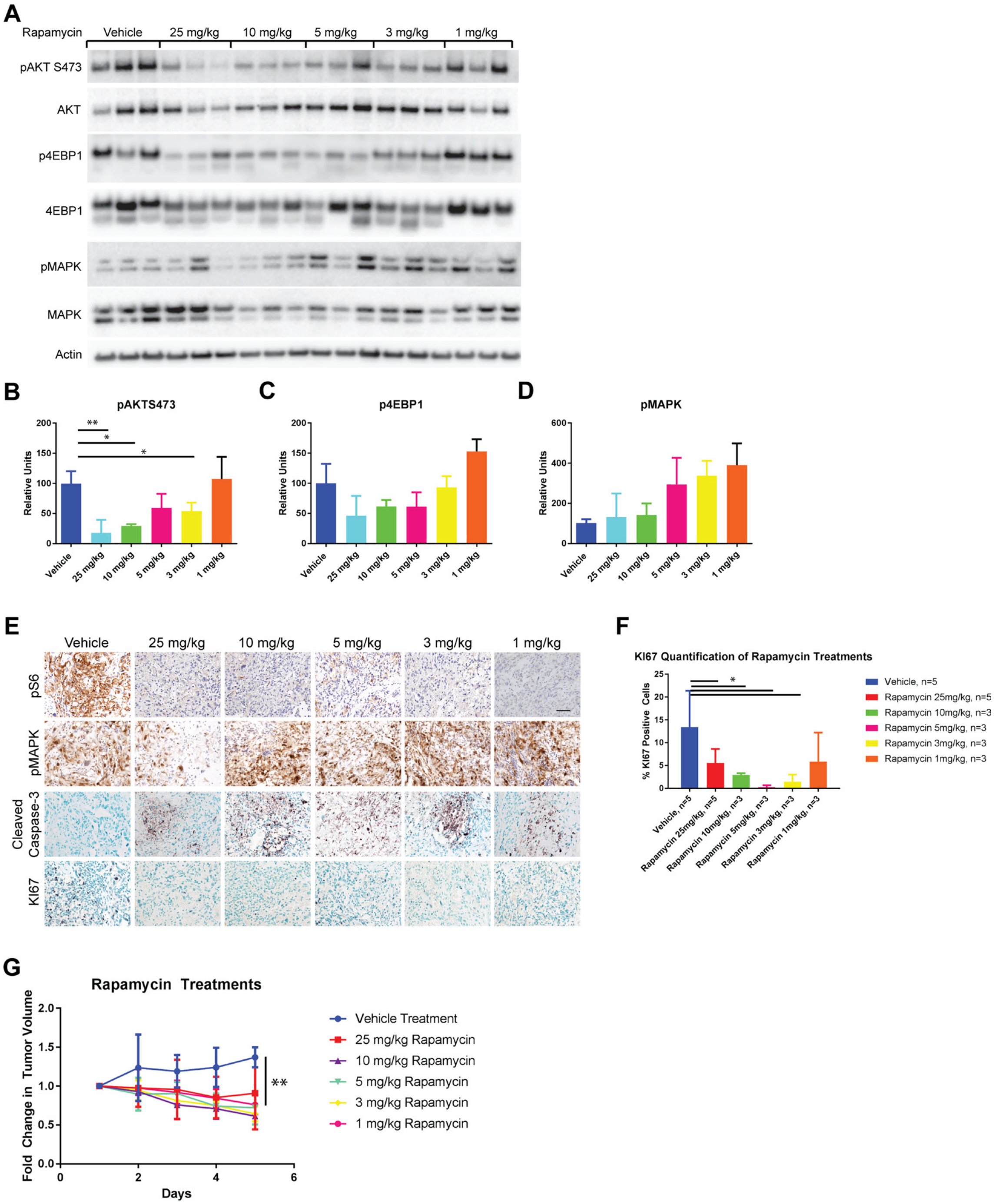

Supplementary Figure 3: Dose response of rapamycin against angiosarcoma. (**A**) Western blots of signaling molecules in protein lysates prepared from tumors of vehicle-treated TKO mice compared with rapamycin-treated TKO mice after five days of treatment at 25 mg/kg, 10 mg/kg, 5 mg/kg, 3 mg/kg, and 1 mg/kg. (**B–D**) Quantification of the indicated phosphoproteins from the western blots (A). $^{*}P \leq 0.05$, $^{**}P \leq 0.01$. (**E**) IHC on representative tumors from mice treated as indicated above demonstrating activity of mTOR and MAPK pathways, apoptosis, and proliferation. Scale bar in top right panel represents 50 µm and applies to all panels. (**F**) Quantification of KI67 from (E). $^{*}P \leq 0.05$ applies to all comparisons indicated. (**G**) Change in tumor volume in mice treated with different drug concentrations over the course of five days of treatment. Vehicle n = 6, 25 mg/kg n = 8, 10 mg/kg n = 3, 5 mg/kg n = 3, 3 mg/kg n = 3, 1 mg/kg n = 3. $^{**}P \leq 0.01$ applies to comparisons between vehicle and each concentration.

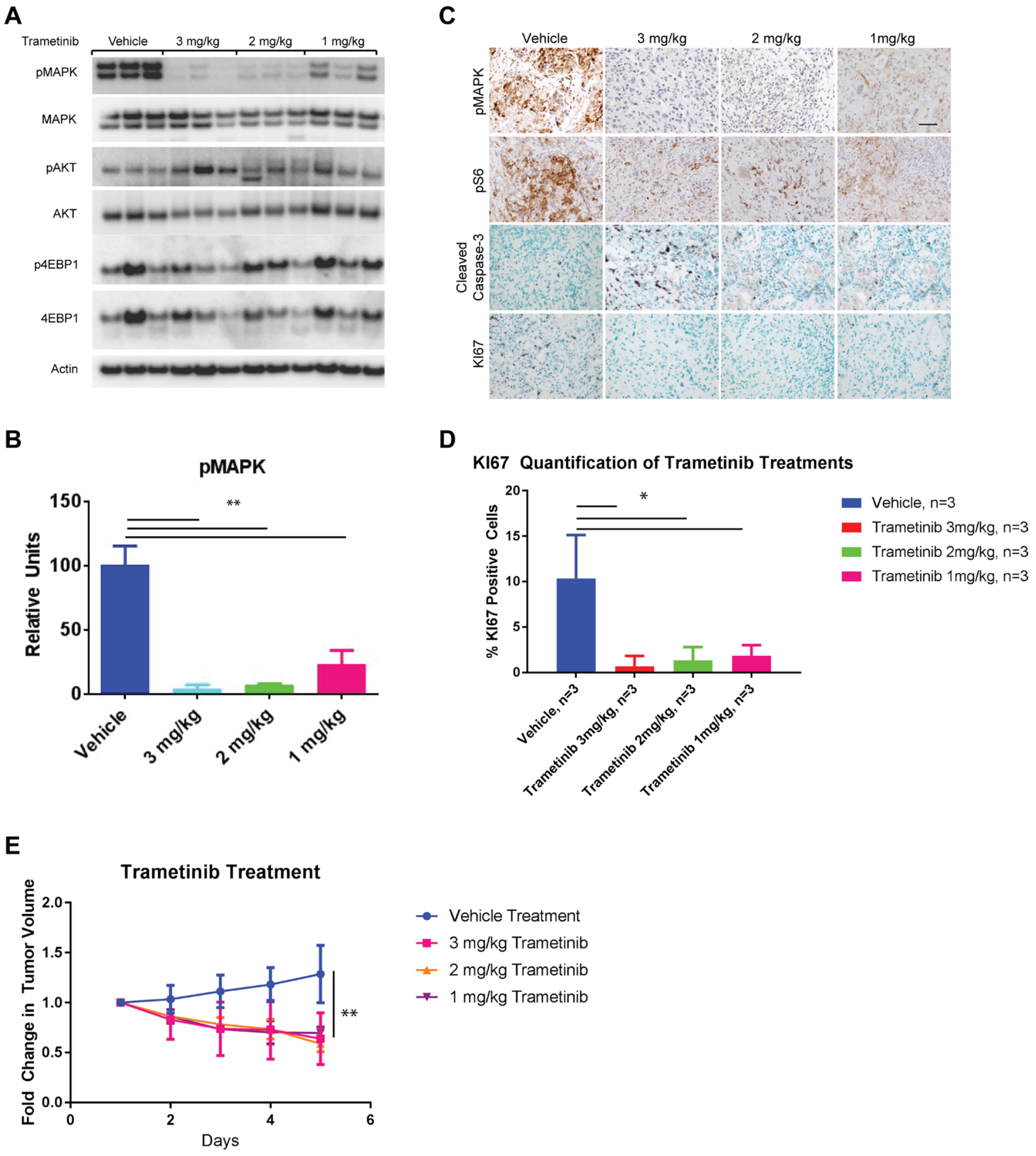

Supplementary Figure 4: Dose response of trametinib against angiosarcoma. (**A**) Western blots of signaling molecules in protein lysates prepared from tumors of vehicle-treated TKO mice compared with trametinib-treated TKO mice after five days of treatment at 3 mg/kg, 2 mg/kg, and 1 mg/kg. (**B**) Quantification of pMAPK from the western blots in (A). $^{**}P \leq 0.01$ and applies to all comparisons indicated. (**C**) IHC on representative tumors from mice treated as indicated above demonstrating activity of mTOR and MAPK pathways, apoptosis, and proliferation. Scale bar in top right panel represents 50 µm and applies to all panels. (**D**) Quantification of KI67 from the IHC in (C). $^{*}P \leq 0.05$ and applies to all comparisons indicated. (**E**) Comparison of change in tumor volumes between the different drug concentrations and the vehicle control. Vehicle $n = 7$, 3 mg/kg $n = 8$, 2 mg/kg $n = 3$, 1 mg/kg $n = 3$. $^{**}P \leq 0.01$ applies to comparisons between vehicle and each concentration.

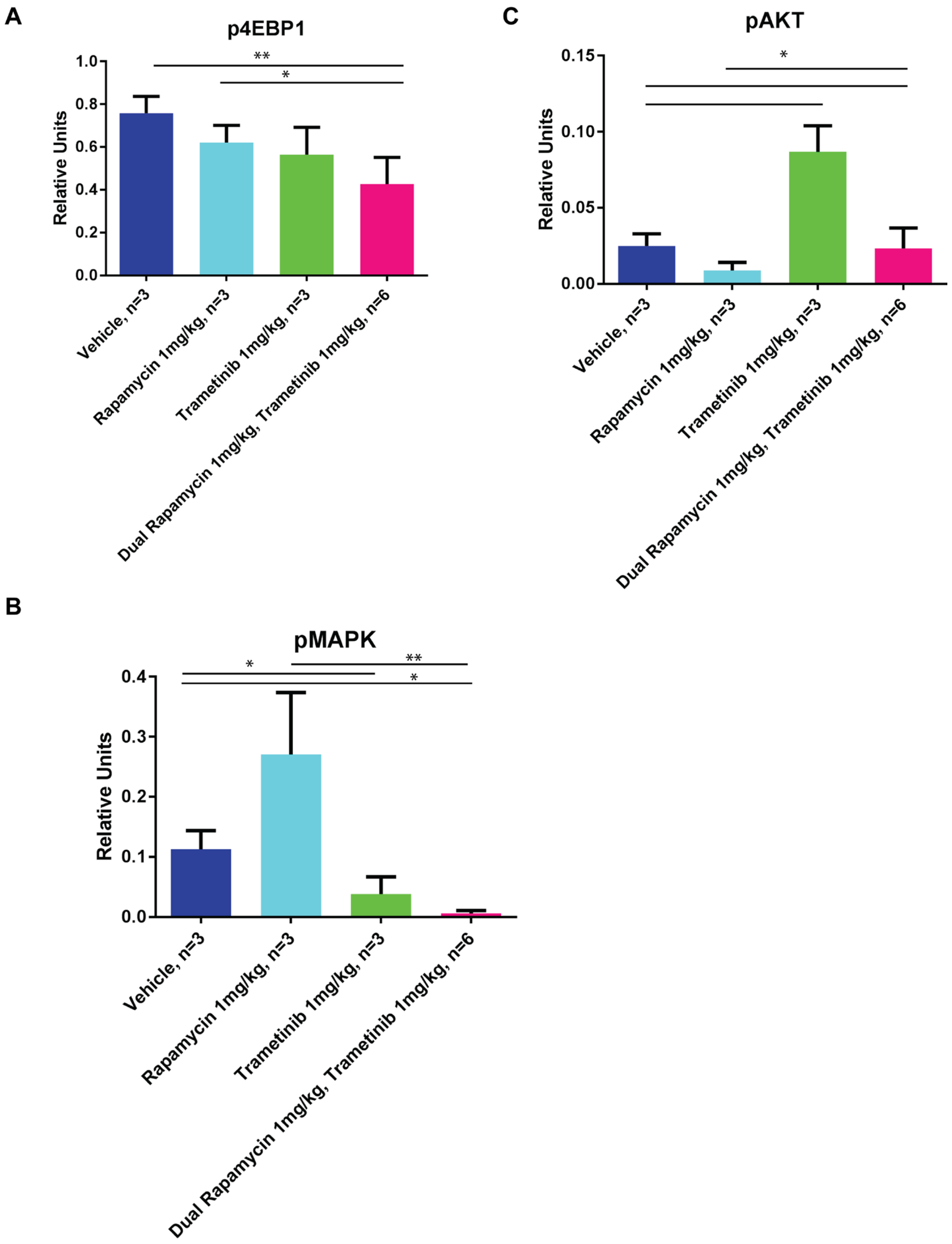

Supplementary Figure 5: Combination therapy results in best inhibition of mTOR and MAPK signaling pathways. Quantification and statistical significance for the western blots in Figure 3A. (**A**) Quantification of p4EBP1 from the western blots showing improved inhibition with dual drug treatment. $^{*}P \leq 0.05$, $^{**}P \leq 0.01$. (**B**) Quantification of pMAPK from the western blots showing greater inhibition with dual drug treatment. $^{*}P \leq 0.05$, $^{**}P \leq 0.01^{**}$. (**C**) Quantification of pAKT Ser473 from the western blots showing greater inhibition with dual drug treatment and significant hyperphosphorylation in trametinib-treated tumors. $^{*}P \leq 0.05$ applies to all comparisons indicated.

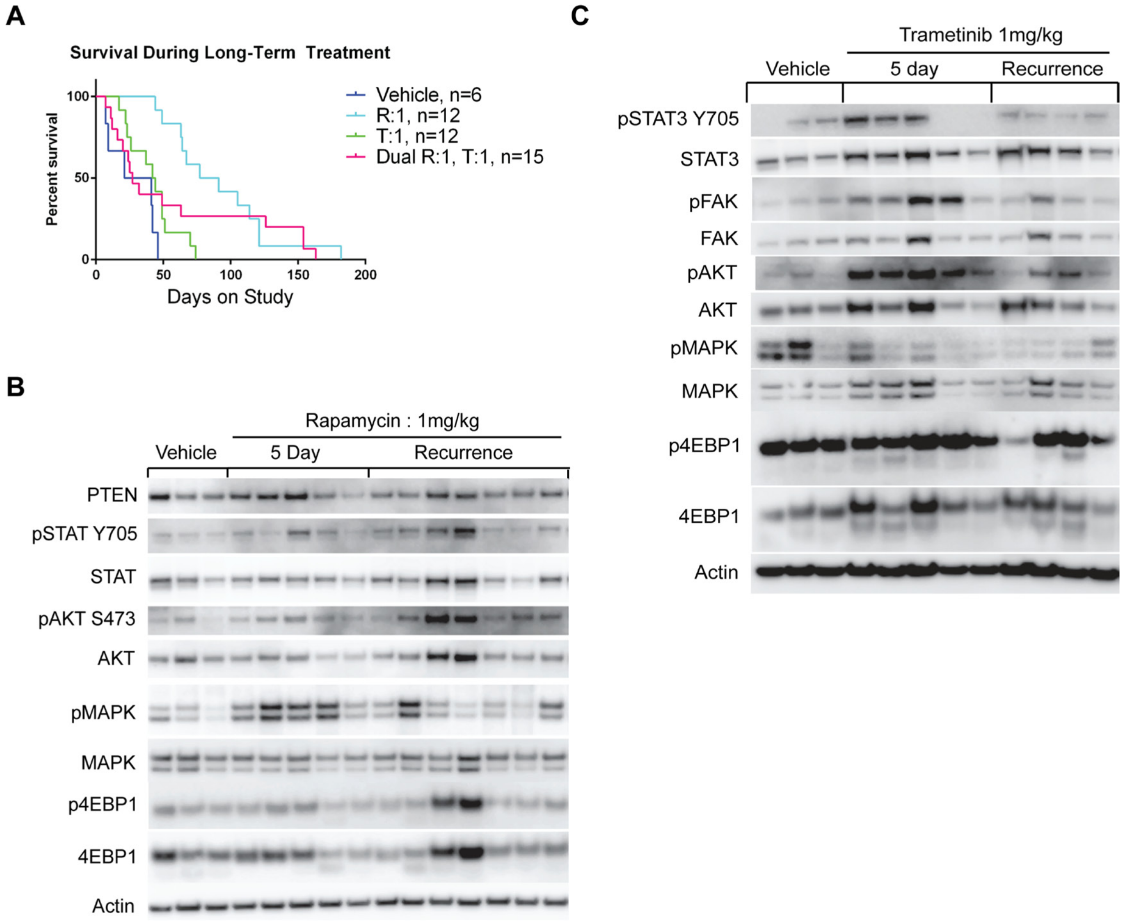

Supplementary Figure 6: Rapamycin or trametinib treatment leads to tumor recurrence long term; mice treated with both drugs experience drug toxicities. (**A**) Kaplan-Meier analysis of overall survival of TKO mice on long-term drug treatments at 1 mg/kg. Dual treated mice die largely due to drug toxicities rather than disease progression (Figure 4B). (**B**) Western blots of protein lysates from rapamycin-treated tumors at 5 days or recurring on long-term treatment compared with vehicle. (**C**) Western blots of protein lysates from trametinib-treated tumors at 5 days or recurring on long-term treatment compared with vehicle.

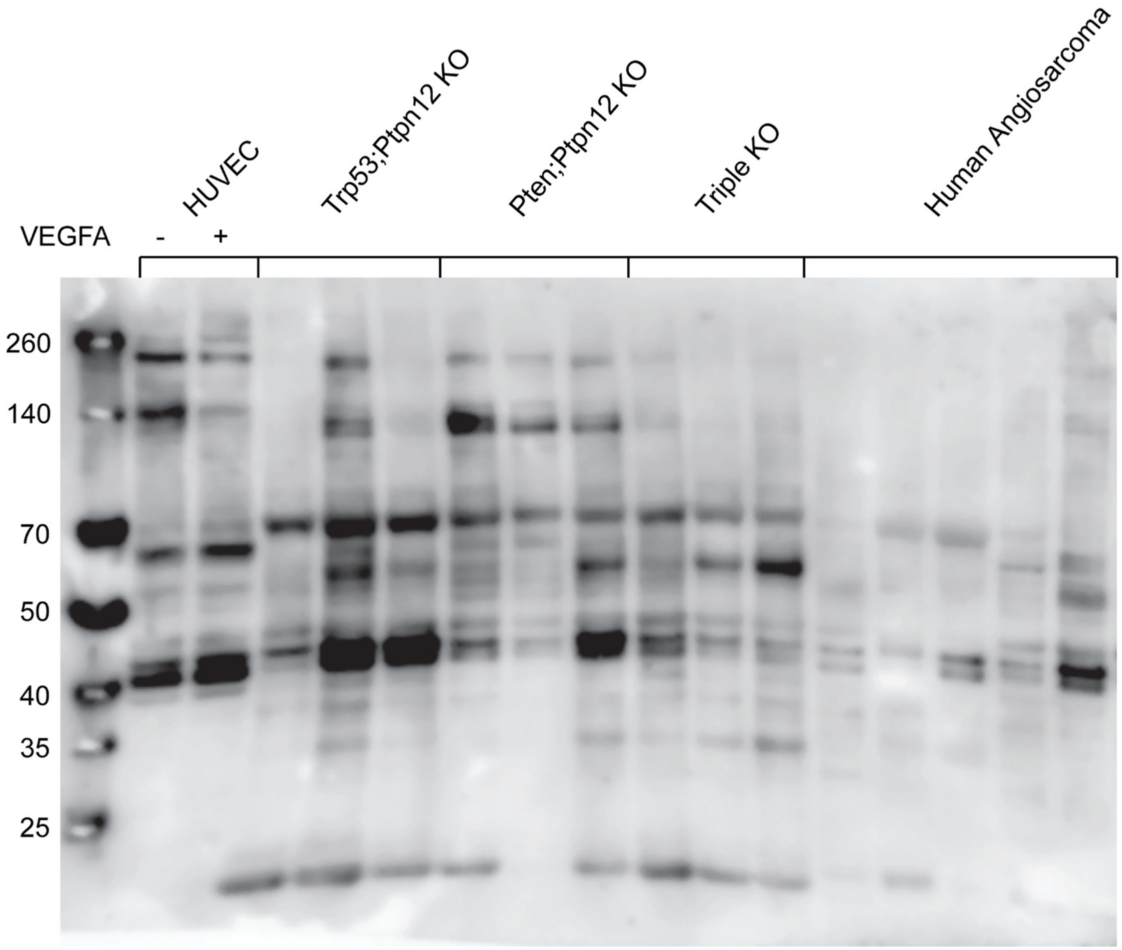

Supplementary Figure 7: Proteins around 40 and 70 kd are tyrosine phosphorylated in human and murine angiosarcomas. Anti-phosphotyrosine western blot of protein lysates prepared from HUVECs treated (+) or untreated (–) with VEGFA, angiosarcomas from all three mouse genotypes, and a panel of human angiosarcomas.

Supplementary Table 1: Index of all RTKs represented on the array and their position

Mouse Phospho-RTK Array					
**Coordinate***	**Receptor Family**	**RTK/Control**	**Coordinate***	**Receptor Family**	**RTK/Control**
A1, A2	Reference Spots	-	C17, C18	TIE	TIE-1
A23, A24	Reference Spots	-	C19, C20	TIE	TIE-2
B1, B2	EGFR	EGFR	C21, C22	NGFR	TRKA
B3, B4	EGFR	ERBB2	C23, C24	NGFR	TRKB
B5, B6	EGFR	ERBB3	D1, D2	NGFR	TRKC
B7, B8	EGFR	ERBB4	D3, D4	VEGFR	VEGFR1
B9, B10	FGFR	FGFR2 (IIIc)	D5, D6	VEGFR	VEGFR2
B11, B12	FGFR	FGFR3	D7, D8	VEGFR	VEGFR3
B13, B14	FGFR	FGFR4	D9, D10	MuSK	MuSK
B15, B16	Insulin R	Insulin R	D11, D12	EPHR	EPHA1
B17, B18	Insulin R	IGF-1R	D13, D14	EPHR	EPHA2
B19, B20	AXL	AXL	D15, D16	EPHR	EPHA3
B21, B22	AXL	DTK	D17, D18	EPHR	EPHA6
B23, B24	AXL	MER	D19, D20	EPHR	EPHA7
C1, C2	HGFR	HGFR	D21, D22	EPHR	EPHA8
C3, C4	HGFR	MSPR	D23, D24	EPHR	EPHB1
C5, C6	PDGFR	PDGFRα	E1, E2	EPHR	EPHB2
C7, C8	PDGFR	PDGFRβ	E3, E4	EPHR	EPHB4
C9, C10	PDGFR	SCFR	E5, E6	EPHR	EPHB6
C11, C12	PDGFR	FLT-3	E7, E8	Control (-)	PBS
C13, C14	PDGFR	M-CSFR	F1, F2	Reference Spots	-
C15, C16	RET	c-RET			

*array coordinates correspond to row letter (A–F) and column number (1–24).
